# Building microbial communities to improve antimicrobial strategies

**DOI:** 10.1038/s44259-025-00115-1

**Published:** 2025-05-30

**Authors:** Laurence Couture, Fabrice Jean-Pierre, Jean-Philippe Côté

**Affiliations:** Département de biologie, Université de Sherbrooke, Sherbrooke, Québec, J1K 2R1 Canada

**Keywords:** Drug screening, Microbial communities

## Abstract

The lack of novel antimicrobial compounds in the development pipeline cries for innovative approaches regarding their discovery. In this Perspective, we discuss how microbial interactions play a significant role in shifting a pathogen’s response to antibacterial treatment and negatively impact patient outcomes. Furthermore, we argue that interspecies interactions are often overlooked in treatment selection and current drug screening approaches, and modeling disease-relevant polymicrobial communities could help in unraveling novel strategies to eradicate pathogens.

Between the 1940s and 1960s, the rate at which new antibiotics were discovered raised hopes of easily treating bacterial infections for years to come. However, within just a few decades, traditional approaches regarding their discovery unfortunately appear to have reached a standstill as our antibiotic pipeline remains limited in new options. Furthermore, the global rise of antimicrobial resistance (AMR) and tolerance mechanisms observed during hard-to-treat infections emphasizes the pressing need for innovative strategies to revitalize antibiotic discovery.

Changes in antimicrobial susceptibility of microbes grown as polymicrobial communities have been reported since the 1960s^1,2^. However, to this day, current treatment strategies aimed at eradicating infections mostly adhere to Koch’s “one microbe, one disease” postulate^3^. Although extensive literature exists on the mechanisms by which microbes escape antimicrobial eradication in the context of pure bacterial culture^4^, the impact of microbial interactions on antimicrobial response is typically undervalued. Herein, we discuss how interspecies interactions can exacerbate infection severity, leading to treatment failure, and present a series of observations that strongly support the need to revisit our current approaches aimed at identifying novel compounds to improve treatment success.

## The importance of microbial interactions in driving infections

Several human infections are polymicrobial in nature^5,6^. For example, chronic cystic fibrosis (CF) lung disease—which is driven by genetic mutations in a single gene coding for an ion channel resulting in abnormal mucus production in the airway—and chronic non-healing wounds (CWs), are well-characterized diseases where multiple pathogens coexist and are difficult to eradicate given their recalcitrance to therapy^6^. Indeed, clinical features of both CF lung disease and CWs are often negatively impacted by polymicrobial communities. For example, multiple studies indicate that the presence of numerous bacterial taxa correlates with reduced CF lung function and antibiotic treatment failure in CWs compared to individuals colonized by a singular microbe, translating into worsened patient outcomes^7,8^.

One of the main causes hypothesized to impact disease progression and treatment failure is microbial interactions. That is, bacteria are often found in heterogeneous communities that play a critical role in exacerbating disease through the establishment of complex networks driven by microbe-microbe interactions^6^. For example, mechanisms driving such interactions can include (but are not limited to): (i) metabolic cross-feeding, (ii) quorum sensing signals, (iii) production of secondary metabolites, (iv) exchange of genetic material, and (v) chemical or enzymatic reshaping of the surrounding environment^9^. These polymicrobial events can likely contribute to disease progression by influencing various aspects of bacterial physiology, ultimately worsening patient outcomes^6^. Of clear clinical relevance, the presence of more than one species during infection—such as *Pseudomonas aeruginosa* and *Staphylococcus aureus*—can result in treatment failure compared to individuals colonized by one microbe^10^.

The interaction between *P. aeruginosa* and *S. aureus* is probably one of the most studied co-culture model given their high prevalence and abundance in various disease types and their clinical relevance^11–13^. Of note, *P. aeruginosa* can increase *S. aureus* tolerance to vancomycin—a front-line antibiotic—and their co-occurrence can result in worsened outcomes in patients^9^. This interspecies dynamic also translates to the genetic level, where a change in essentiality of over 200 genes of *S. aureus* is observed in co-culture^14^. In a murine lung infection model, the presence of *P. aeruginosa* also led to a proportional increase in *S*. *aureus* airway colonization^15^. In the human gastrointestinal tract, proximity between trillions of microbes also gives rise to a complex network of interactions that are crucial for pathogen colonization^16^. Conversely, pathogens also interact with commensals of the digestive tract to establish themselves at the infection site^16,17^. Like *P. aeruginosa*, interactions between intestinal pathogens and the gut microbiota could potentially impact the susceptibility of the pathogen to antibiotics. Supporting this, *Salmonella enterica* serovar Typhimurium undergoes changes in its metabolism when co-cultured with lactobacilli strains, which translates to an increased tolerance against azithromycin, an antibiotic used to treat salmonellosis^18^. At the oral cavity level, microbial interactions are known to underlie many oral diseases (e.g., cariogenic plaque), notably through the establishment of polymicrobial biofilms^19^. For example, the symbiotic interaction within plaque biofilms of *Candida albicans* and *Streptococcus mutans* leads to increased virulence of the biofilm and results in a more severe form of infection^20^. Taken together, the above-mentioned examples highlight the importance of microbial interactions in exacerbating various disease types.

## Current treatment limitations for polymicrobial infections

Although we are beginning to appreciate the importance of polymicrobial communities during disease, the current gold standard used to guide antibiotic selection heavily relies on antimicrobial susceptibility testing (AST). However, such an approach depends on the ability of a molecule to inhibit the growth of a unique microbe and may explain why such a strategy oftentimes does not readily translate into treatment success^21,22^. For example, retrospective studies have shown that AST-based approaches fail to inform antibiotic-based clinical decisions aimed at eradicating *P*. *aeruginosa* and *S*. *aureus*^8,23^. Therefore, multiple reports advocate that AST in its current form (i.e., relying on the growth inhibition of a single bacterium in pure culture) has fallen short and should not be used to guide antibiotic selection to treat polymicrobial-based infections^21,22,24^. Unfortunately, clinical evidence aimed at guiding treatment selection during chronic and polymicrobial-based infections is still lacking and oftentimes biased towards specific pathogens. For example, while >70% of pulmonary exacerbation events are polymicrobial, a recent study including >4000 people with CF (pwCF) highlighted a strong clinical bias toward exclusively treating *P. aeruginosa* during CF airway infections^25^. More worryingly, Faino and colleagues reported that antibiotic treatments used during prior exacerbation events are more likely to be reselected in a future event, even though different and less conventional pathogens are detected^25^. With increasing evidence showing that microbes respond differently when grown in a polymicrobial environment compared to pure culture, current AST-based methods need to be revisited to better reflect the complex microbial composition detected at the site of infection.

While the above-mentioned examples indicate that interactions between at least two microbes can impact infection severity, one could argue that using antimicrobials aimed at eradicating these pathogens simultaneously could result in positive disease outcomes. However, such a strategy does not seem to be as straightforward as one would think. For instance, in a retrospective study including >900 children with CF who were positive for co-colonization with *P. aeruginosa* and methicillin-resistant *S. aureus* (MRSA), combining antipseudomonal antibiotics with anti-MRSA therapy did not result in improved outcomes compared to monotherapy^26^.

Other factors that can influence bacterial susceptibility to antibiotics and, in turn, affect the accuracy of AST include phenotypic and genotypic variations within species and environmental conditions to which bacteria are exposed to^24^. As traditional AST is often performed in nutrient-rich media, an effective way of assessing the actual efficacy of a compound against a pathogen is through the development of growth culture media that reflect the conditions of the infection site^27^. Growing knowledge on the nutritional composition found at various body sites has led to the development of multiple disease-mimicking media, such as the artificial urine medium (AUM), synthetic synovial fluid media and synthetic cystic fibrosis medium (SCFM2), which are commonly used to simulate in vitro the human urine, human joint fluid and CF environment, respectively^28–31^. Promoting bacterial aggregation, a synthetic synovial fluid medium modeling essential features of the periprosthetic joint infection (PJI) environment, induces a decreased susceptibility of various common PJI pathogens to antibiotics compared to traditional media^30^.

Taken together, we posit that the emergence of community-intrinsic phenotypes in infection-relevant conditions may help elucidate why many promising compounds are effective at inhibiting growth when tested against a single bacterium but fail to do so when used in real clinical settings where the bacterium resides within a polymicrobial community^24^. Furthermore, we suggest that it might be possible that some compounds lacking activity in monoculture become potent in a polymicrobial context^9,13,32,33^. For example, chloroxylenol (an antiseptic) has poor activity against *S. aureus* monocultures, but is significantly more effective when *S. aureus* is exposed to *P. aeruginosa* metabolites^32^. Norfloxacin, a quinolone antibiotic, also shows increased potency against biofilms of *S. aureus* in the presence of *P. aeruginosa* exoproducts^33^. However, while such examples show exciting potential applications, we first need an improved and unbiased understanding of microbial heterogeneity and interactions observed during infection and, ultimately, a better-tailored approach for treating such infections.

## Building model communities to understand microbial interactions

As highlighted by O’Toole in a recent editorial, to better understand how a given microbe interacts with others in its environment, we first have to start by defining groups of microbes that can serve as models to investigate the microbial dynamics in the community^33^. These model communities would then fulfill a similar role to model microbes, which is to provide valuable knowledge about microbial systems that can be applied to more complex ones. Most reported examples of microbe-microbe interactions altering the phenotype of a pathogen are co-culture experiments involving two microbes. However, using co-culture model systems begs the question “Are two microbes enough?” and, more importantly, “How many should we use to model interactions?”. While we argue that there is not a straightforward answer to these questions, it is worth noting that all steps toward replicating as closely as possible the polymicrobial environment that pathogens encounter during infection seem like the logical progression to revitalize antibacterial drug discovery. One would first have to determine which questions the model is aiming to answer and then build its community around relevant microbial features required to tackle them^34,35^. To understand the molecular basis underlying a community-emergent phenotype, it is also important to consider that the microbial consortium should be simplified to a degree that allows for easy characterization of the community dynamics. The goal is not necessarily to have a model that perfectly mirrors the in vivo community, but rather a model that is better tailored (or at least as close as possible) for addressing the specific questions at hand.

Several strategies can be employed to design a microbial community to serve various research purposes. The community that has been the focus of most modeling efforts in recent years is probably the gut microbiota. Multiple synthetic microbial communities of the gut have been designed using a bottom-up (i.e., increasing model complexity) approach, which consists of selecting bacterial candidates based on desired functions^36^. For example, the Oligo-Mouse-Microbiota (OMM^12^) is a well-established model composed of twelve bacterial species that mimics the functional and compositional traits of the murine gut microbiota and confers colonization resistance against enteric pathogens, notably toward *S*. Typhimurium^37^. Measurement of microbial taxa abundances in clinical samples of pwCF also allowed the elaboration of an in vitro community to study the dynamics among co-existing dominant bacteria in the CF lung^38^. This community is composed of *P. aeruginosa*, *S. aureus*, *Streptococcus sanguinis* and *Prevotella melaninogenica*—which together account for ≥70% of 16S rRNA gene reads detected in ~62% of analyzed patient samples. When grown in this community, *P. aeruginosa* was more susceptible to tobramycin, a clinically relevant antibiotic for treating chronic CF lung infections, compared to monoculture^38^. More importantly, the same study defined the mechanism associated with the recalcitrance of a common variant of *P. aeruginosa* prevalent in the CF airway. Taken together, these infection models could potentially represent an untapped potential to serve as disease-relevant systems for the identification of compounds with novel antimicrobial properties.

## Using model communities in infection-relevant conditions to drive drug discovery and impact human health

Since the beginning of antibacterial drug discovery, our screening methods have been performed under a pure-culture framework using nutrient-rich microbiological media^39^. However, we now recognize that growth conditions during infection differ significantly from this context^40^. As gene essentiality can significantly differ depending on strains and experimental context, and only a small subset of genes is essential for the growth of bacteria in nutrient-rich media, this led to a portfolio of clinically available compounds that target a limited number of essential cellular processes, mainly cell-wall, protein, and DNA synthesis^41^. Transposon mutagenesis studies performed during infection have been conducted for several pathogens and have shown that the number of essential genes in vivo greatly exceed the ones detected during in vitro growth in rich media^42–44^. Thus, by only targeting in vitro essential processes, we are omitting many biological functions that are also essential for pathogens during an infection.

Capturing the CF environment, SCFM2 is now widely used to study the physiology and pathogenesis mechanisms of *P*. *aeruginosa* during chronic CF lung infections. Interestingly, growing *P. aeruginosa* in SCFM2 was found to more accurately capture genes of this microbe expressed in CF sputum compared to growth in lysogeny broth (LB) and even a mouse model^45^. In short, growing *P. aeruginosa* in SCFM2 resulted in a genome-wide accuracy score of 85.9%—meaning that 85.9% of *P. aeruginosa* genes using SCFM2 were similarly expressed compared to what is seen directly in sputum, whereas the mouse and LB models had an accuracy of 80.6% and 80.4%, respectively. This difference in accuracy represents 300 additional genes that are captured by SCFM2 but not accounted for by the other models. Such approaches are not limited to CF lung infections. For instance, a new medium mimicking the intestinal environment also proved to better capture both functional and compositional profiles of the gut microbiota, therefore acting as an excellent in vitro gut microbiome model^46^.

Unsurprisingly, these “infection-like” media are now being used in the search for new antibacterial compounds and have allowed for the identification of new active compounds that are often overlooked using traditional approaches. A screen looking for compounds preventing the growth of *Klebsiella pneumoniae* in serum has uncovered many compounds with increased activities in this condition^47^. One of these compounds, Ruthenium red, could specifically decrease the bacterial burden in blood in a systemic infection model of *K. pneumoniae*. Similarly, comparative screening of a pre-approved drug library in rich media compared to artificial sputum medium (ASM) has identified molecules with increased activities against *P. aeruginosa* specifically in ASM^48^. Adding mammalian cells to probe intracellular pathogens also represents a strategy to discover new compounds. For example, combining other model types (mice, cell lines) with SCFM2 can help in increasing the accuracy of the models and capture elusive genes that would not be well-represented by growing *P. aeruginosa* in SCFM2 alone^49^.

**Fig. 1:**
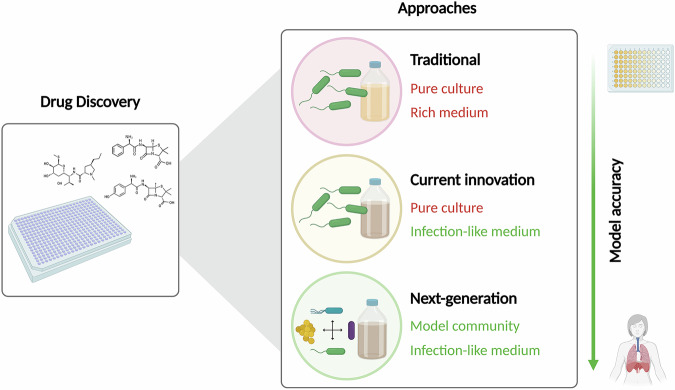
Improving current drug discovery strategies to combat microbial-based infections. Traditional models have generally used a pure culture approach in rich medium to pinpoint compounds capable of eradicating pathogens observed at the site of infection. While such strategy has driven the success of antibiotic discovery, integrating disease-relevant parameters have allowed to fine-tune our current approaches to increase their innovation potential and find compounds with novel antimicrobial activities. We propose that we should go even further by modeling a community of microbes relevant to a disease to integrate microbe-microbe interactions in future drug discovery screening strategies and thus improve the chances of eradicating hard-to-treat polymicrobial infections. Created with Biorender.

While using media that mimic the infection environment is a great step toward innovating drug discovery approaches, bacterial interactions are still overlooked in our efforts to bridge the gap between the laboratory and the clinic. Thus, we propose that for polymicrobial-associated infections, experimental efforts aimed at modeling these communities should be made and used to catalyze even more innovation in our drug discovery approaches (Fig. 1). Such a strategy holds great promise in unraveling new ways to target pathogens that will translate to positive clinical results. By combining a model community in an “infection-like” medium with a mutant library of a pathogen, for example, we could comprehensively investigate the genetic requirements of a microbe to interact with neighboring species found at its infection site. To support such applications, proper consideration should be given to the microbes that constitute the model community to keep it tractable and amenable to high-throughput frameworks. As microbial interactions can give rise to unexpected features of bacterial physiology, we posit that the implementation of model communities at an early stage of the drug discovery process could support the repurposing of compounds that are already approved for other clinical contexts, and lead to a significant leap in the identification of compounds with new modes of action. Redirecting our drug screening efforts toward the target of genes or cellular processes involved in microbial interactions could drive a level of innovation in current traditional screening approaches that is critically needed, considering the fulgurant increase in AMR.

## Data Availability

No datasets were generated or analysed during the current study.
